# Virtual dissection of *Aedes aegypti* mosquito using phase‐contrast synchrotron microtomography

**DOI:** 10.1111/jmi.70004

**Published:** 2025-06-23

**Authors:** Gabriela Sena, Thaina Alvarenga, Ruan Ingliton Feio, Cícero Brasileiro Mello, Gabriel Fidalgo, Katrine Paiva, Tayane Tanure, Liebert Parreiras Nogueira, Marcos Vinícius Colaço, Arissa Pickler, Marcelo Salabert Gonzalez, Patricia Azambuja, Giuliana tromba, José Bento Pereira Lima, Ademir Xavier da Silva, Regina Cély Barroso

**Affiliations:** ^1^ Laboratory of Applied Physics to Biomedical Sciences, Physics Institute, State University of Rio de Janeiro Rio de Janeiro Brazil; ^2^ Nuclear Engineering Department COPPE, Federal University of Rio de Janeiro Rio de Janeiro Brazil; ^3^ Post‐Graduate Program in Science and Biotechnology, Federal University Fluminense Rio de Janeiro Brazil; ^4^ Oral Research Laboratory (ORL) Institute of Clinical Dentistry University of Oslo Oslo Norway; ^5^ SYRMEP Elettra‐Sincrotrone Trieste SCpA Trieste Italy; ^6^ Laboratory of Biology, Control and Surveillance of Insect Vectors Oswaldo Cruz Institute, FIOCRUZ Rio de Janeiro Brazil

**Keywords:** aedes aegypti, microtomography, phase‐contrast

## Abstract

In this paper, in‐line phase‐contrast synchrotron microtomography was used to study the morphology of adult *Aedes aegypti*. These specimens are vectors of several arboviruses, causing dengue, chikungunya, Zika and yellow fever. The morphological details of this insect species are still incomplete and insufficient. To address this gap, this study examined whole specimens of *Aedes aegypti* in the adult phase at high resolution. For this, the adult samples were scanned in the microtomography beamline (SYRMEP) at the Italian Synchrotron Light Laboratory (ELETTRA). The phase‐contrast technique allowed us to obtain high‐quality images, which made it possible to evaluate the segmentation of structures on the rendered volume by the Dragonfly software. The combination of high‐quality images and segmentation process provide adequate visualisation of different organs which could serve in assessing the effectiveness of innovative control population methods as a basis for future control studies of the insect vector.

## LAY DESCRIPTION

This study utilized in‐line phase‐contrast synchrotron microtomography to investigate the morphology of adult Aedes aegypti, a vector of several arboviruses such as dengue, chikungunya, Zika, and yellow fever. Despite the importance of this species, its morphological details remain incomplete. To address this, the researchers scanned whole adult specimens at high resolution using the microtomography beamline (SYRMEP) at the Italian Synchrotron Light Laboratory (ELETTRA). The phase‐contrast technique provided high‐quality images, enabling the segmentation of structures. This approach allowed for detailed visualization of various organs, which could aid in evaluating innovative population control methods for future studies on vector control.

## 1 INTRODUCTION

The *Aedes aegypti* are the main vectors of several arboviruses, responsible for the dengue, Zika, yellow fever and chikungunya. A recent study has shown that there are 215 countries potentially suitable for the survival and establishment of *Aedes aegypti* and globally, 146 countries reported at least one arboviral disease.^1^

Millions of dollars are spent annually to eradicate these arboviral diseases. Until now, there is no effective remedy against these arboviruses, so disease prevention can be done in two ways. First one is the reduction of mosquito infestations, a measure that has been promoted and carried out in recent years by research centres in some countries and, second, by the use of an effective vaccine. However, there are only vaccines available for yellow fever and dengue. For other arboviruses, the most effective method of eradication remains vector control.^2^, ^3^

Despite the need for innovative control techniques, studies about the morphology of *Aedes aegypti* mosquitoes remain incomplete.^4^ Visualisation in detail of the internal and external structures of *Aedes aegypti* may be of great importance in vector control.^5^, ^6^, ^7^, ^8^, ^9^, ^10^ One example is the fact that there are many researches aiming to describe the effectiveness of essential plant oils plants for *Aedes aegypti* mosquito vector control. Detailed visualisation of the insect's morphology could be a valuable tool in these studies, helping to understand the effects of these essential oils on the mosquito's structures.^11^, ^12^

In this study, we describe the protocol used to visualise the morphology of *Aedes aegypti* male using Phase‐Contrast Synchrotron Radiation Microtomography (PC‐SR‐MicroCT). The main advantage of this technique is that it does not require the dissection of the insects, significantly reducing samples loss and damage during preparation.^13^, ^14^ The experiments were performed at SYRMEP beamline, ELETTRA, Trieste. The protocol developed in this work will serve as a basis for future insect control studies.

## 2 MATERIALS AND METHODS

### 2.1 Creation and maintenance of larvae of *Aedes aegypti*

In this study, *Aedes aegypti* of the Rockefeller strain was used. The eggs were provided by the Laboratory of Physiology and Control of Vector Arthropods – LAFICAVE of the Oswaldo Cruz Foundation – FIOCRUZ, located at the Army Biology Institute – IBEX.

The eggs were laid to hatch in water at 30°C. After 60 min, first instar larvae were transported to a 1L plastic tray containing a proportion of 1 mg of fish food (Tetramin®) in 1 mL of water for each larva. Finally, the tray covered with net was kept in BOD incubator at 26 ± 1°C until metamorphosis. Only newly emerged male adults were used in the experiments.

The mosquitoes were refrigerated for approximately 15 min at 4°C to ensure they were inert for handling. Subsequently, the most morphologically intact individuals were selected and grouped in sets of five. Using entomological forceps, the insects were carefully transferred to 2.0 mL polypropylene tubes (Microtube type) containing 1.5 mL of Bouin's fixative (1:1 in water). After 4 h of incubation at room temperature, the specimens were transferred and stored in tubes containing 1.5 mL of 70% ethanol until further analysis.

### 2.2 Methods

The In‐line phase‐contrast microtomography scans were performed using the set‐up available at the SYRMEP beamline of ELETTRA synchrotron facility (Trieste, Italy).^15^ In order to optimise the performances of the microtomography set‐up for high‐resolution experiments, a lens‐coupled CCD camera system designed to achieve up to a pixel size of 0.9 µm was used in white X‐ray beam mode. The sample‐to‐detector distance was set to 15 cm and the average energy was around 16 keV. For our experiments, 1800 radiographic projections were acquired over an angular range of 180° with angular steps of 0.1°. Tomographic slices were reconstructed via conventional filtered back‐projection algorithm using the SYRMEP TOMO PROJECT (STP) software developed by the SYRMEP team.^16^ In this work, the approach to the inverse problem of phase retrieval based on transport‐of‐intensity (TIE) algorithm^17^ was used. Image processing and reconstruction of the 3D volume were carried out using Dragonfly software.^18^

### 2.3 Results

Studying the morphology of the *Aedes aegypti* mosquito is of great importance in understanding its biology and developing effective control strategies. The intricate structure of this vector species provides critical insights into its behaviour, physiology, and interactions with its environment.^1^, ^2^

Although pathogen transmission is closely related to female *Aedes aegypti* mosquitoes due to the process of haematophagy,^19^ it is noteworthy that males actually emerge before females.^20^ This phenomenon, known as protandry, is well‐documented in the literature.^21^, ^22^ It occurs due to the need for sexual maturation of the males before copulation, which may confer an advantage by increasing their opportunities for mating.^23^, ^24^

Thus, the study of male anatomy is of significant relevance, as it provides a deeper understanding of the biology of the male insect. This knowledge can be applied to the development of vector control strategies, such as the Sterile Insect Technique (SIT), which focuses exclusively on the release of sterile males. The release of non‐viable males directly impacts the number of reproductively active females, thereby reducing the mosquito population, as the offspring from such matings will be inviable.^25^

**FIGURE 1:**
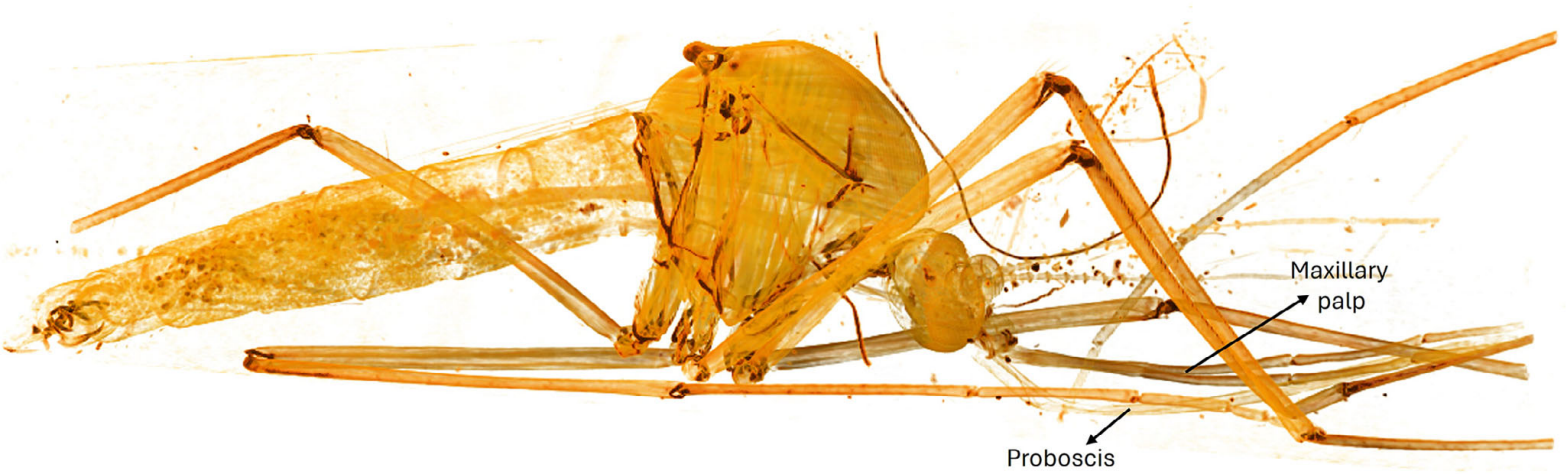
3D rendering of an *Aedes aegypti* male obtained using phase‐contrast synchrotron microtomography. The identification as male is confirmed by the presence of reproductive organs exclusive to males, including testes and accessory glands, as shown in Figures 5 and 6. External structures such as the maxillary palps, which are as long as the proboscis in males, are also visible.

The results revealed intricate details of various organs and important structures of the mosquito. Figure 1 displays a three‐dimensional image of an entire *Aedes aegypti* male, obtained using phase‐contrast synchrotron microtomography. This image showcases the comprehensive visualisation capabilities of this technique.

**FIGURE 2:**
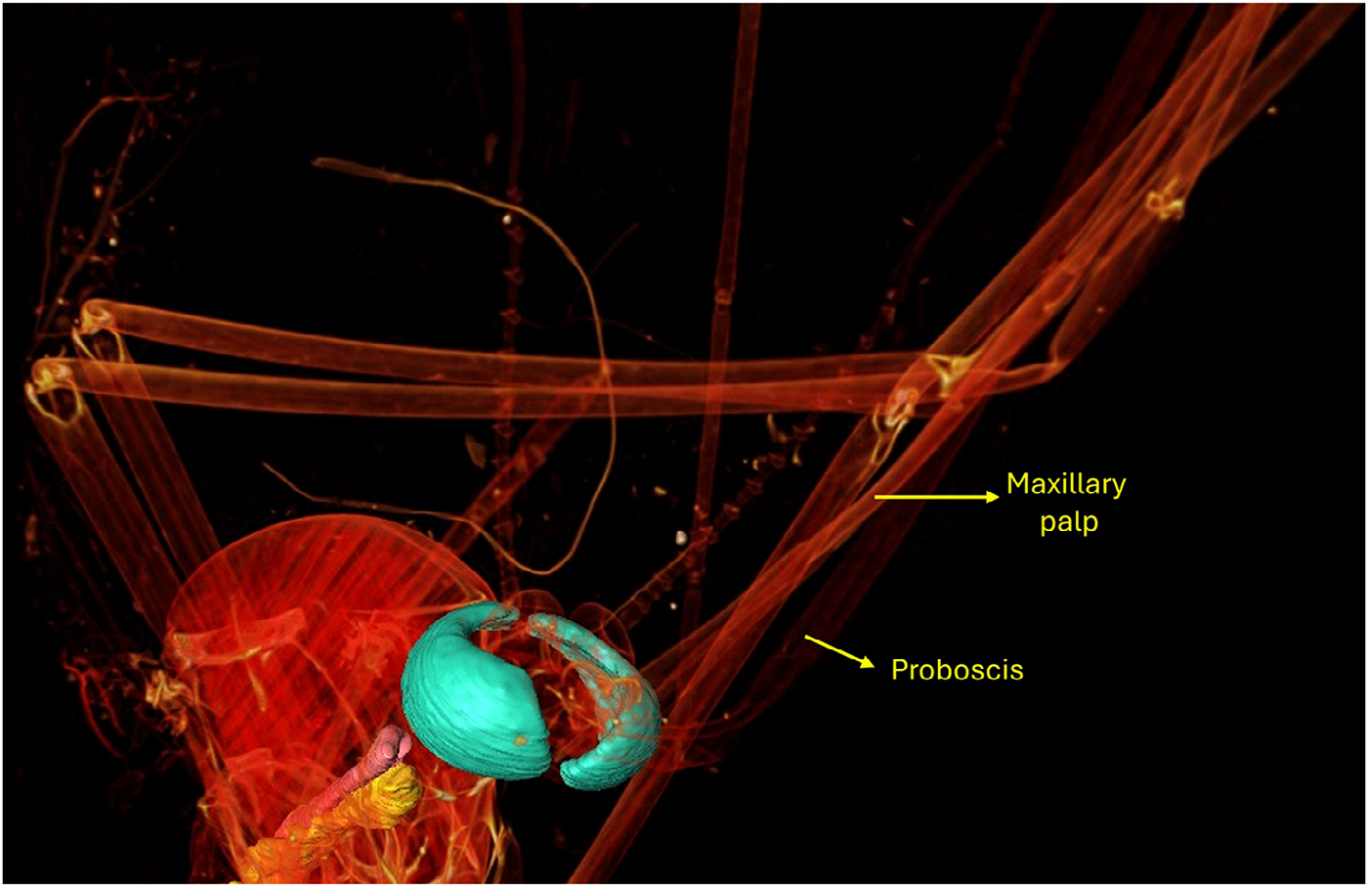
Detailed 3D visualisation of the compound eyes of the same *Aedes aegypti* male specimen. These eyes are essential for spatial orientation, host‐seeking behaviour, and mating.

Among the structures that distinguish male from female *Aedes aegypti*, the maxillary palps are external structures that allow for the identification of the mosquito's sex. According to the comparison presented by Andrew,^26^ the maxillary palps in males are as long as the proboscis, whereas in females, the maxillary palps are shorter in comparison to the proboscis^27^ and Bohbot.^28^ These structures can be identified in Figures 1 and 2.

**FIGURE 3:**
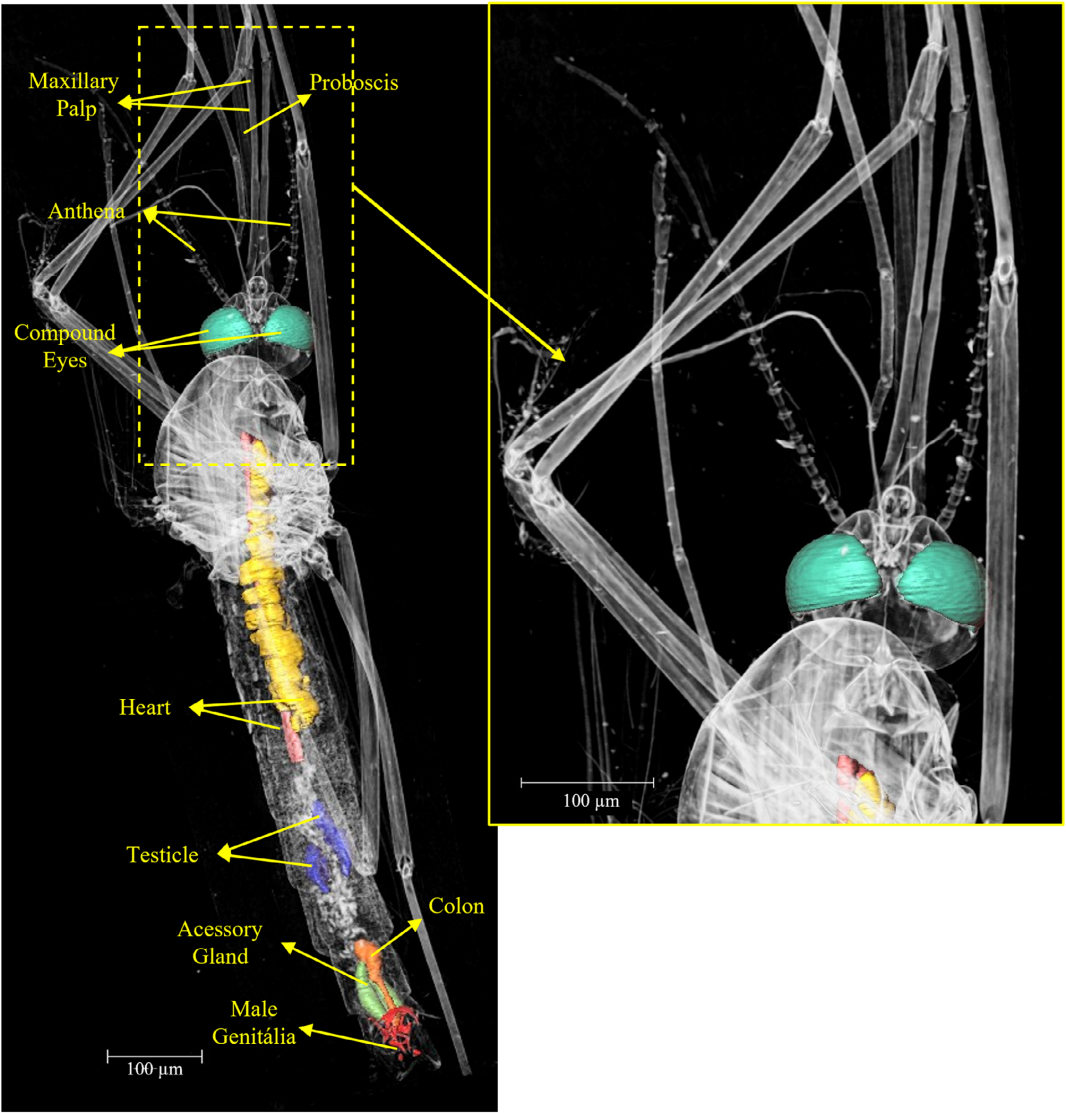
Alternative 3D perspective of the compound eyes of the male specimen, highlighting their complex surface structure.

Figures 2 and 3 show the compound eyes of the *Aedes aegypti* mosquito, a structure that plays a pivotal role in its survival and ability to transmit diseases. These organs contribute significantly to the mosquito's overall behaviour, physiology, and reproductive success.^29^

The compound eyes of the *Aedes aegypti* are specialised for detecting visual cues, allowing the mosquito to locate potential hosts for blood feeding and identify suitable breeding sites. The sensitivity of their eyes to different wavelengths of light helps these mosquitoes navigate their surroundings with precision, enabling them to adapt to diverse environments.^30^

Understanding the visual capabilities of the *Aedes aegypti* is crucial for the development of effective control strategies. Research on the visual processing pathways in their brains can unveil vulnerabilities that may be targeted to disrupt their ability to find and feed on human hosts. Additionally, insights into their visual preferences can inform the design of traps or attractants that mimic human‐related visual stimuli, offering innovative approaches for mosquito surveillance and control.

Furthermore, the eyes of the *Aedes aegypti* are involved in crucial behaviours such as courtship and mating. Studying the intricate mechanisms behind their visual communication can provide valuable information for the development of strategies aimed at interfering with mosquito reproduction.

**FIGURE 4:**
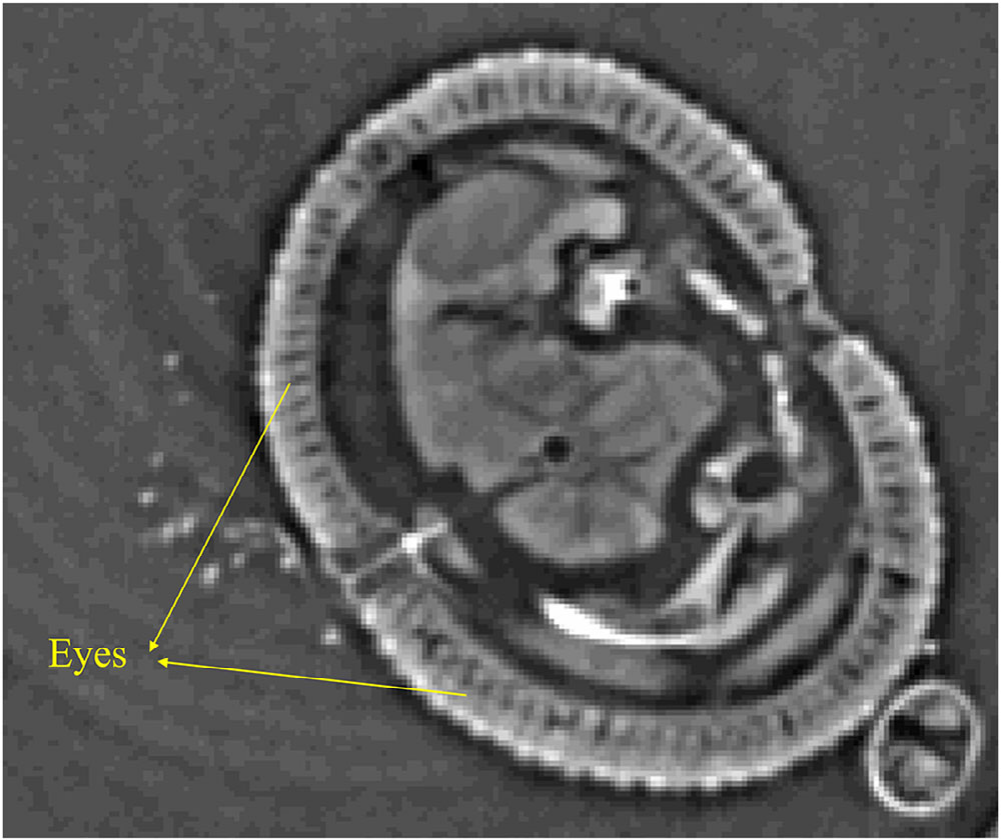
Virtual transversal slice of the male *Aedes aegypti*, showing the internal anatomy of the compound eyes and adjacent tissues.

In summary, the eyes of the *Aedes aegypti* mosquito are not merely sensory organs; they are key players in the mosquito's life cycle and disease transmission dynamics. A comprehensive understanding of their visual ecology is essential for devising targeted and sustainable approaches to mitigate the impact of these mosquitoes on public health.^31^ Figure 4 shows a transversal virtual cut used to segment the compound eyes structures.

**FIGURE 5:**
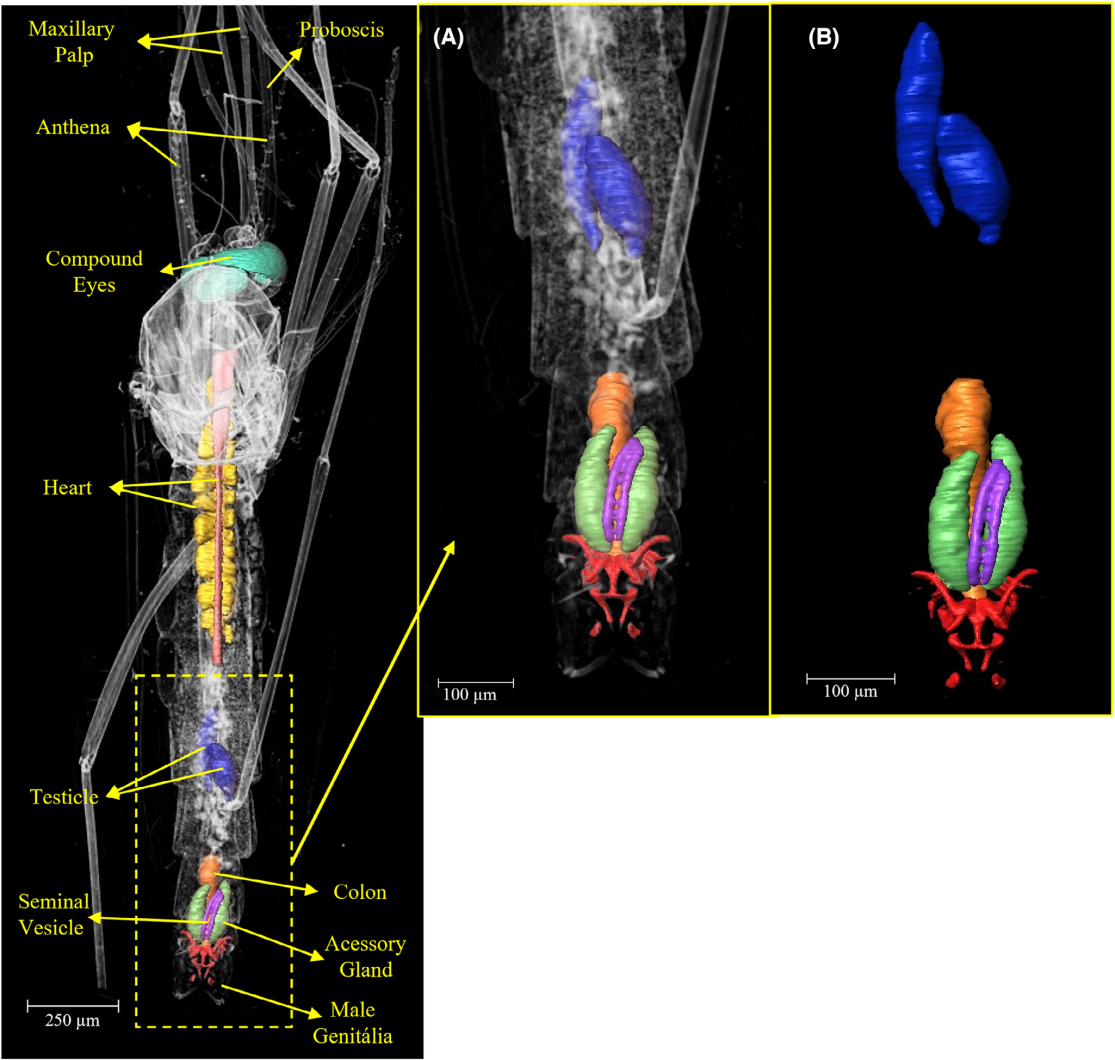
(A, B) 3D segmentation of key internal organs in the *Aedes aegypti* male specimen. Highlighted structures include testes (blue) – responsible for sperm production; male accessory glands (green) – produce seminal fluid; colon (orange) – terminal part of the digestive tract; genitalia (red) – external reproductive structure of the male.

Figure 5A and B shows the accessory glands, colon and testicles of the *Aedes aegypti* male mosquito, which are crucial components of its physiology, contributing significantly to its ability to transmit diseases. Investigating the glands of male *Aedes* mosquitoes may uncover biochemical pathways or pheromones that play key roles in mating behaviours. This knowledge could offer opportunities to more effectively disrupt transmission cycles of vector‐borne diseases.^32^

The colon, or hindgut, of the *Aedes aegypti* is a vital organ involved in the final stages of digestion and water reabsorption. Understanding the dynamics of this organ is essential for comprehending the mosquito's feeding behaviour and optimising the development of control measures. By investigating the colon, researchers can identify vulnerabilities in the digestive process that may be exploited for the development of novel larvicides or inhibitors, disrupting the mosquito's ability to mature and reproduce.^33^

Furthermore, the accessory glands and colon are integral components in the context of disease transmission. The mosquito's ability to efficiently feed on human hosts and subsequently transmit pathogens is closely linked to the proper functioning of these organs. Investigating the molecular and physiological mechanisms governing these processes is crucial for devising targeted interventions that impede the transmission of diseases like dengue and Zika.

Studying the testicles of the *Aedes aegypti* is crucial for understanding its reproductive mechanisms, such as sperm production and maturation processes.

**FIGURE 6:**
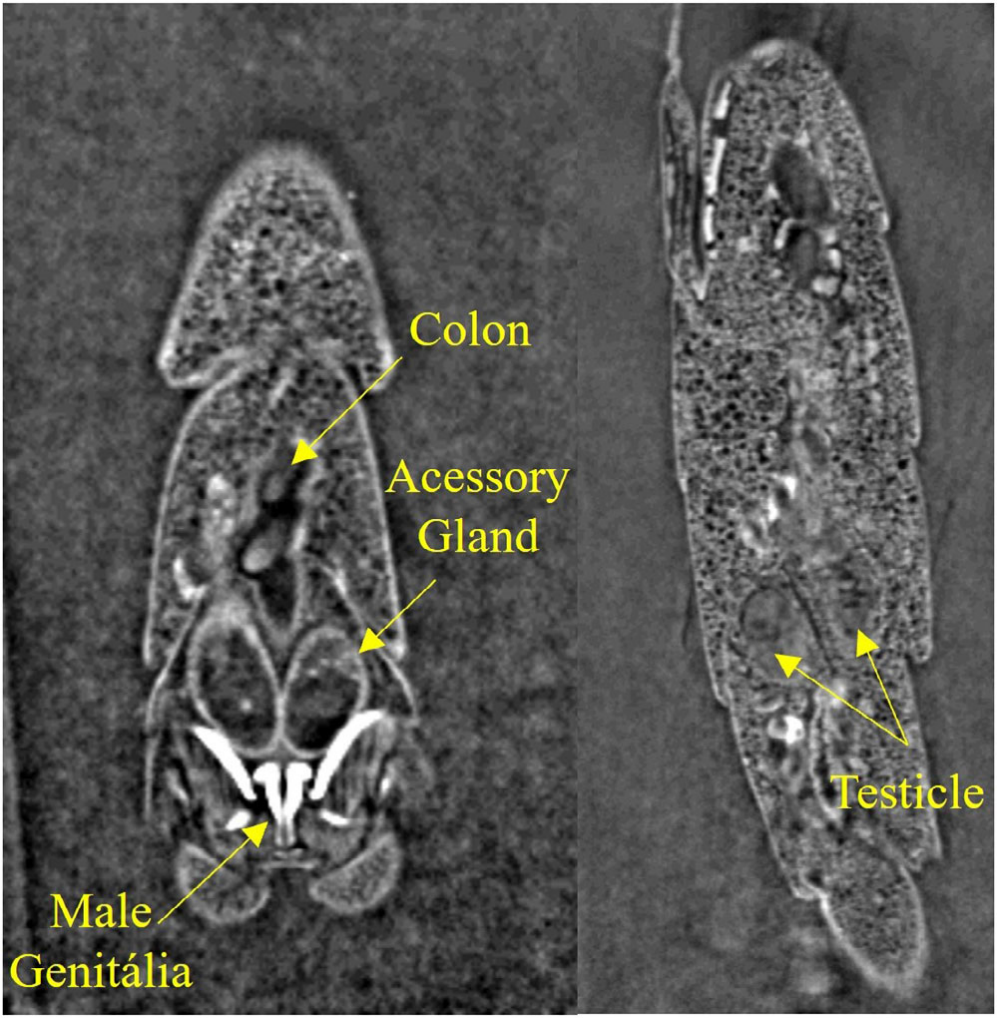
Representative virtual slice showing the segmentation of the testes, male accessory glands, and colon in the male *Aedes aegypti* specimen. This image confirms the sex of the specimen through the presence of male‐exclusive reproductive organs.

The accessory glands and colon of the *Aedes aegypti* mosquito are not only key elements in its reproductive and digestive processes but also represent potential targets for innovative control strategies. By delving into the intricacies of these organs, researchers can contribute to the development of sustainable and effective approaches to mitigate the impact of mosquito‐borne diseases on global public health.^34^ Figure 6 shows a section of one of the slices used for the glands and colon segmentation.

**FIGURE 7:**
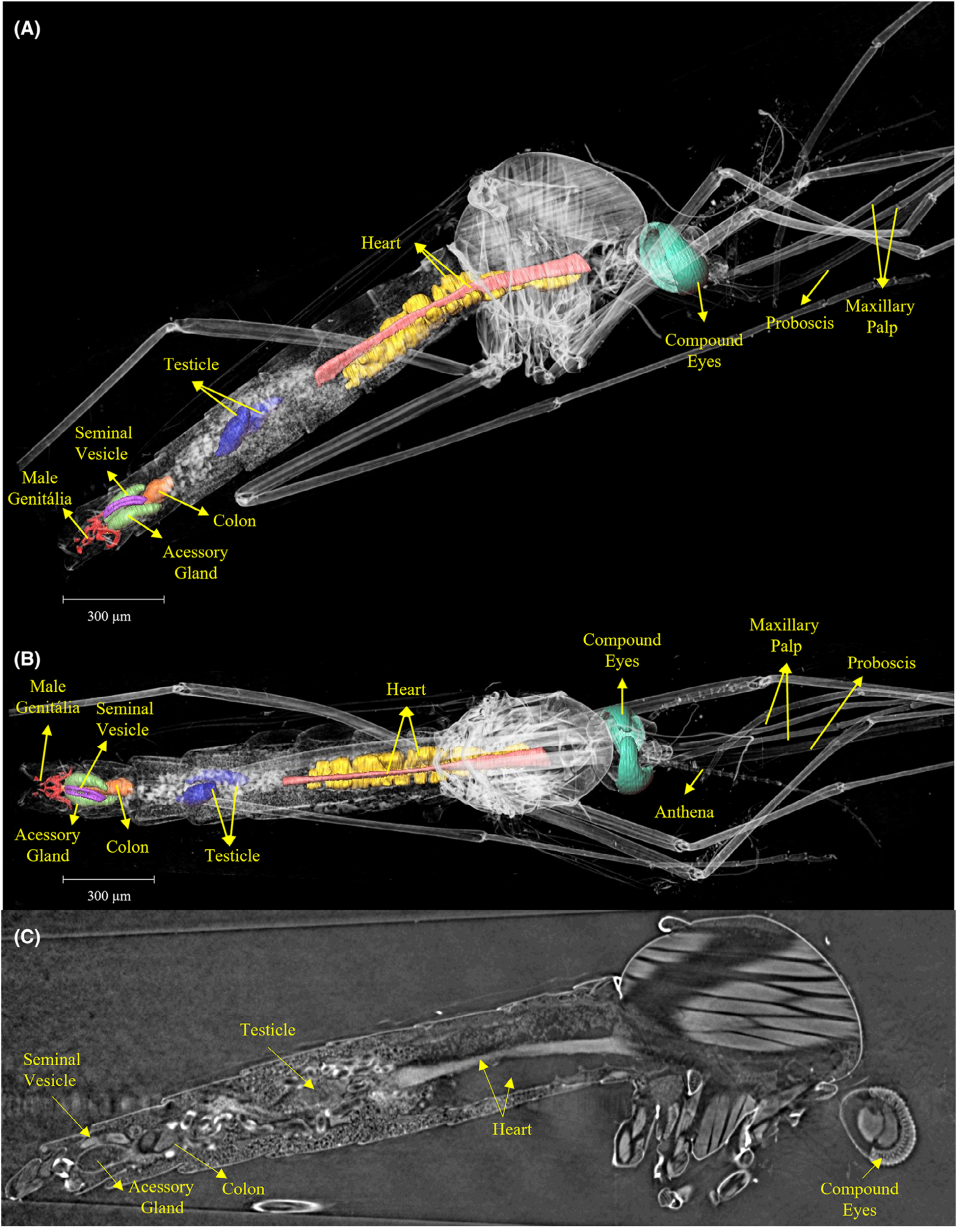
(A–C) 3D visualisation of the heart (yellow) and haemocytes (pink) in the male *Aedes aegypti*, along with the compound eyes (green). The heart circulates haemolymph throughout the body, playing a critical role in nutrient transport and pathogen dissemination.

In Figure 7A–C, it can be seen mainly the heart of the *Aedes aegypti*, which plays a crucial role in its survival and reproductive success. It pumps haemolymph, the insect equivalent of blood, throughout its body, delivering essential nutrients and oxygen. This circulation also facilitates the spread of pathogens like dengue, Zika, and chikungunya viruses, making the heart a pivotal factor in disease transmission. Understanding its physiology is key to developing effective strategies for mosquito control and combating the diseases they carry.

## 3 CONCLUSION

The *Aedes aegypti* mosquito is a vector for several devastating diseases, such as dengue, Zika, and chikungunya. While much attention is often focused on its role in disease transmission, understanding the importance of specific organs, such as the glands, colon and compound eyes are crucial for devising effective control strategies.

The study of the mosquito's morphological adaptations can inform the design of innovative control measures. From developing more effective traps and attractants to understanding the vulnerabilities in their exoskeleton and internal anatomy, a detailed examination of the *Aedes aegypti's* morphology provides the foundation for developing sustainable and targeted approaches for mosquito control.

Although this study focuses on the dissection of males and does not directly address pathogen transmission, the detailed analysis of the internal morphology of male *Aedes aegypti* mosquitoes could support future interventions aimed at controlling and reducing the vector population. This underscores the importance of males in the mosquito's life cycle and their indirect role in controlling diseases transmitted by females. Furthermore, future research will provide a detailed examination of the reproductive structures of females, emphasising their significance in pathogen transmission. Thus, a combined analysis of both males and females could offer a more comprehensive understanding of the biology of *Aedes aegypti* and its implications for disease control.

Our study utilised the SYRMEP beamline at ELETTRA, where the application of phase‐contrast synchrotron microtomography proved crucial in resolving the soft tissues of *Aedes aegypti* mosquitoes. The protocol used significantly enhances and facilitates sample preparation by eliminating the need for dissection, thus preserving the integrity of the samples. Additionally, the imaging regime at the beamline was optimised to achieve detailed visualisation of both internal and external structures, demonstrating the technique's potential for future research in mosquito‐borne disease control.

In conclusion, the results obtained from synchrotron phase‐contrast microtomography, along with extensive segmentation of some target organs and structures revealed the detailed morphology of the *Aedes aegypti*. This serves as a blueprint for understanding its biological intricacies and developing strategies to mitigate its impact on public health. Through a comprehensive exploration of its structural adaptations, researchers can pave the way for innovative and environmentally conscious approaches to mosquito‐borne disease control. The protocol established in this study will serve as a model for future insect control studies.
